# Targeting Cell Death: Pyroptosis, Ferroptosis, Apoptosis and Necroptosis in Osteoarthritis

**DOI:** 10.3389/fcell.2021.789948

**Published:** 2022-01-18

**Authors:** Jian Yang, Shasha Hu, Yangyang Bian, Jiangling Yao, Dong Wang, Xiaoqian Liu, Zhengdong Guo, Siyuan Zhang, Lei Peng

**Affiliations:** ^1^ Trauma Center, The First Affiliated Hospital of Hainan Medical University, Hainan Medical University, Haikou, China; ^2^ Key Laboratory of Emergency and Trauma Ministry of Education, Hainan Medical University, Haikou, China; ^3^ Hainan Provincial Biomaterials and Medical Device Engineering Technology Research Center, Hainan Medical University, Haikou, China; ^4^ Department of Pathology, Hainan General Hospital, Hainan Medical University, Haikou, China

**Keywords:** pyroptosis, ferroptosis, apoptosis, necroptosis, osteoarthritis, molecular mechanism, signal pathway

## Abstract

New research has shown that the development of osteoarthritis (OA) is regulated by different mechanisms of cell death and types of cytokines. Therefore, elucidating the mechanism of action among various cytokines, cell death processes and OA is important towards better understanding the pathogenesis and progression of the disease. This paper reviews the pathogenesis of OA in relation to different types of cytokine-triggered cell death. We describe the cell morphological features and molecular mechanisms of pyroptosis, apoptosis, necroptosis, and ferroptosis, and summarize the current research findings defining the molecular mechanisms of action between different cell death types and OA.

## Introduction

Cell death plays a key role in the development of the body and maintains homeostasis to prevent the development of diseases. Classically, apoptosis and necrosis were viewed as the main types of cell death, however, this paradigm continues to evolve (Walker et al., 1988). Cell death can be defined as programmed and non-programmed forms based on the regulation of the processes involved.

Programmed cell death (PCD) can be divided into lytic and non-lytic cell death (Jorgensen et al., 2017). Non-lytic cell death mainly refers to apoptosis which can produce apoptotic bodies that are cleared by phagocytes and does not involve the inflammatory response (Fuchs and Steller, 2011). Lytic forms of cell death include necroptosis and pyroptosis (Christofferson and Yuan, 2010; Jorgensen and Miao, 2015). These forms of cell death lead to leakage of intracellular components including damage-associated molecular pattern molecules (DAMPs) which in turn activate a strong inflammatory response also known as inflammatory death (Jorgensen and Miao, 2015).

Non-programmed cell death (Non-PCD) generally refers to necrosis which describes the process of irreversible cell damage and final cell death caused by physical or chemical stimulation in extreme environments (Majno and Joris, 1995). The main characteristics of necrosis include the destruction of cell membranes, edema of cells and organelles (cytoplasmic vesicles), and the release of cell contents, however, chromatin does not agglutinate during necrosis (Dondelinger et al., 2017).

A new type of cell death termed ferroptosis, was proposed by Stockwell in 2012 (Dixon et al., 2012). Ferroptosis is a form of programmed cell death driven by iron-dependent lipid peroxidation. The ferroptosis is characterized by changes in the mitochondrial phenotype, mitochondrial atrophy, and an increase of membrane density (Dixon and Stockwell, 2014; Cao and Dixon, 2016). Different types of cell death have been shown to regulate the development of multiple chronic diseases (Zhou et al., 2016; Anderton et al., 2020).

Osteoarthritis (OA) is a chronic degenerative disease with progressive features. OA involves structures all parts of the joints in which undergo structural damage and functional imbalances occur as a result of multiple factors (Loeser et al., 2016). The influence of different types of cell death on the development of OA has become a new research hotspot (Hosseinzadeh et al., 2016; Loeser et al., 2016). The purpose of this review is to summarize the pathogenesis of OA and to explore research opportunities focusing on mechanisms of cell death including pyroptosis, apoptosis and ferroptosis.

### Osteoarthritis

OA is a common and complex chronic disease that affects 250 million people worldwide (Hunter and Bierma-Zeinstra, 2019). Due to an aging population and a rise in obesity, OA has become the fourth largest disabling disease in the world (Woolf and Pfleger, 2003). It is estimated that the medical cost of OA accounts for 1–2.5% of GDP in different high-income countries (Hunter et al., 2014). OA is associated with all parts of the joints and involves structural changes in hyaline articular cartilage, subchondral bone, ligaments, joint capsule, synovium and muscles around the joint (Martel-Pelletier et al., 2016). OA involves metabolic, inflammatory, mechanical and other factors leading to structural damage and repair imbalances. Currently, the main risk factors associated with OA are age, female sex, obesity, hip deformities, weight-bearing work, exercise, diabetes, hypertension, cardiovascular disease, depression and hereditary factors (Hunter and Bierma-Zeinstra, 2019).

Chondrogenic progenitor cells (CPCs) are mesenchymal stem cells that differentiate into chondrocytes and are also known as cartilage precursor cells (Koelling et al., 2009). Chondrocytes and the extracellular matrix are the main components of articular cartilage. No blood vessels or nerves are present in the cartilage matrix, and chondrocytes are the only cellular components present in the articular cartilage matrix. Under physiological conditions, chondrocytes maintain the lowest level of collagen turnover and do not show mitotic activity (Houard et al., 2013). Collagen turnover gradually increases with risk factors such as age, mechanical stress, diabetes and hypertension. Subsequently, the composition and structure of the cartilage matrix change resulting in the formation of fibrous tissue. As this pathological process progresses, deep fissures develop that are related to the shedding of cartilage fragments and eventually lead to the delamination and exposure of calcified cartilage and bone (Hunter and Bierma-Zeinstra, 2019). In the early stages of this process, the surface receptors of chondrocytes inhibit the low collagen turnover rate of chondrocytes through integrins and other related factors. The synthetic activity of chondrocytes also increases significantly due to the repair of the perichondrial matrix which finally develops to invade the collagen network (Xu et al., 2014). This marks the irreversible progression of OA. Also, increases in chondrocyte activity result in the increased production of inflammatory reactive proteins including interleukin-1β (IL-1β), interleukin-6, tumor necrosis factor-α (TNF-α) and matrix metalloproteinases (MMP1, 3 and 13).

Injury to the bone and cartilage around the joint is an important factor in OA. The subchondral bone tissue is composed of cortical and cancellous bone (Burr, 2004; Goldring and Goldring, 2010). As AO progresses, the volume, thickness and outline of the cortical bone gradually increase. Also, the trabecular structure and bone mass of the subchondral bone change as bone cysts form along with the appearance of osteophytes (Goldring, 2008; Xu et al., 2014). A characteristic feature of the OA is the gradual thickening of the subchondral plate that reflects changes in mechanical loading owing to cartilage damage and properties of the subchondral bone (Martel-Pelletier et al., 2016). Shanchez et al., reported that osteoblasts can express inflammatory cytokines and degrading enzymes in response to mechanical stimulation and chondrocytes (Sanchez et al., 2012) that can in turn act on cartilage or subchondral bone to increase the severity of OA.

Synovitis is a common feature of OA (Scanzello and Goldring, 2012). The synovium includes the synovial membrane and the fluid. The synovium is a thin cell layer arranged in the joint cavity that contains macrophages and fibroblasts that regulate the trafficking of molecules through the joint (Shiozawa et al., 2020). The release of inflammatory factors and the secretion of degrading enzymes in the synovial tissue are correlated with the severity of OA (Scanzello and Goldring, 2012). Proteases released by chondrocytes increase the production of pro-inflammatory cartilage degradation products that interact with DAMPs, Toll-like receptors (TLRs) and integrin. These changes further aggravate inflammation and catabolic products to increase the severity of OA (Loeser et al., 2012; Houard et al., 2013; Martel-Pelletier et al., 2016). Baker and Roemer et al. reported that the risk of synovitis progression and OA was positively correlated with joint symptoms through MRI analysis (Baker et al., 2010; Roemer et al., 2011). IL-1, IL-6, IL-15, tumor necrosis factor (TNF), prostaglandin E2, matrix metalloproteinases (MMP1, 3 and 13) and collagenase (coLI, II) have also been detected in the synovial fluid, cartilage and the synovium of patients with OA (Kapoor et al., 2011; Scanzello and Goldring, 2012).

### Pyroptosis

The first experimental observations of lytic death were made over 30 years ago when it was reported that Shigella fowleri could induce lytic death in infected host macrophages. This form of lytic death has characteristics that are common to apoptosis such as chromatin agglutination, DNA fragmentation and cysteinyl aspartate specific proteinase (Caspase) activity dependence (Zychlinsky et al., 1992). This mode of death was initially considered to be apoptosis, yet, it was not until 2001 that Cookson et al. showed that this form of cell death depends on Caspase-1 activity which is different from Caspase-3 activity-dependent apoptosis (Cookson and Brennan, 2001). For the first time, the authors defined pyroptosis as a form of Caspase-1-dependent cell death. The term originates from “pyro” meaning fire, indicating that this kind of programmed cell death causes inflammation, and “ptosis” means falling (Gao et al., 2018), indicating the nature of programmed cell death. When cells undergo pyroptosis, the nucleus becomes concentrated and the chromatin DNA is randomly broken and degraded. Pores in the cell membrane regulate the trafficking of substances to and from cells. As this process becomes imbalanced, osmotic swelling occurs and the membrane breaks (Hersh et al., 1999) releasing the cell contents that contain molecules to stimulate the immune response. During the process of cell death, the nucleus becomes condensed and rounded (Fernandes-Alnemri et al., 2007) yet the nuclear integrity remains unchanged (Santos et al., 2001; Mariathasan et al., 2005).

**FIGURE 1:**
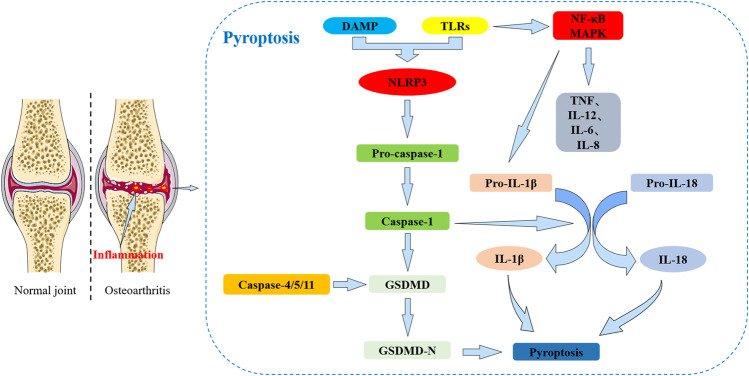
The relationship between pyroptosis and osteoarthritis (OA). Pyroptosis: DAMPs and TLRs can interact to exacerbate the inflammatory response. TLRs initiate a signaling cascade leading to cell activation, increased release of the NLRP3 inflammasome, activation of NF-κB and MAPK signaling pathway, and production of associated inflammatory factors (including TNF, IL-12, IL-6, IL-8 and pro-IL-1β) that in turn activate a strong inflammatory response. The inflammatory response promotes the increased release of IL-1β and IL-18 on the cartilage surface exacerbating cartilage damage and also enhancing pyroptosis signaling. The NLRP3 inflammasome can activate caspase-1 which further activates GSDMD to undergo shearing to form the N-terminal end. The N-terminal end of GSDMD leads to cell membrane perforation and ultimately induces pyroptosis. Caspases-4/5/11 are also able to activate GSDMD and can induce pyroptosis.

Pyroptosis mainly leads to the splicing and polymerization of members of the Gasdermin family (such as GSDMD). It can also activate cell perforation and death through inflammatory corpuscle activation and caspases. Based on the different mechanisms of caspase-mediated cell death, pyroptosis can be defined as caspase-1 and caspase-4/5/11 types (Figure 1). Pyroptosis induced by caspase-1 activation mainly occurs in macrophages and dendritic cells (Fink et al., 2008; Broz et al., 2010). Caspase-4/5/11 activation can also cause pyroptosis in macrophages and other non-macrophage cell types (Kayagaki, Warming et al., 2011).

Gasdermin proteins are a family that has multiple functions and are expressed in a variety of cell types and tissues. Human gasdermin proteins are composed of Gasdermin A (GSDMA), Gasdermin B (GSDMB), Gasdermin C (GSDMC), Gasdermin D (GSDMD), Gasdermin E (GSDME, also known as DFNA5) and Pejvakin (PJVK, also known as DFNB59) (Feng et al., 2018). Except for PJVK, all gasdermin proteins have conserved double domain arrangements including a C-terminal (GSDM-C) and N-terminal domains (GSDM-N). The N terminal has a pore-forming activity and can induce pyroptosis (Yang et al., 2018). GSDMD can be specifically activated by caspases-1, 4, 5, 11, and can be cut by caspase-1 and 11 into GSDMD-N (p30 fragment) and GSDMD-C (p20 fragment) (Zhao L. R. et al., 2018; Kayagaki et al., 2015). The activated GSDMD-N domain has lipophilic characteristics and can be transported from the cytoplasm to the cell membrane (Liu et al., 2016; Shi et al., 2017). The N-terminus of gasdermin can directly interact with membrane lipids and oligomerize to form 10–33 nm pores (Aglietti et al., 2016; Sborgi et al., 2016; Shi et al., 2017). New research has shown that intracellular IL-1β can be released outside the cell through channels formed by GSDMD. When exposed to inflammation or hyperactivating stimuli, GSDMD and caspase-11 can form larger pores in the liposomes of cell membranes leading to a massive release of IL-1β which in turn causes pyroptosis (Evavold et al., 2018). Recently, it has been demonstrated that when GSDME is stimulated by chemotherapeutic agents, tumor necrosis factor (TNF) and viral infection, it is activated by caspase-3 which is involved in apoptotic signaling. GSDME then releases the activated N-terminal end causing perforation of the cell membrane which converts cells that should undergo apoptosis into pyroptosis (Wang et al., 2018). These data further suggest that the regulation of cell death is highly complex and dependent on the mode of death.

The main types of inflammasomes include NLRP3, NLRP1, NLRC4 and AIM2. The basic structure of inflammasomes consists of pattern recognition receptors (PRRs), apoptosis-associated speck-like protein containing a CARD (ASC) and pro-caspase-1 (CASP1) (Man and Kanneganti, 2015; von Moltke et al., 2013). Inflammasomes are multimeric protein complexes that assemble in the cytosol and act as platforms for caspase activation (An et al., 2020). When cells sense inflammation or the invasion of viral microbes, they respond by damaging tissues (Matzinger, 2002). A series of cascade reactions mediated by the TLRs are initiated to activate nuclear factor-κB (NF-κB), mitogen-activated protein kinase (MAPK) and interferon signaling pathways leading to cell activation and the production of related inflammatory cytokines. These include tumor necrosis factor (TNF), interleukin-6 (IL-6), interleukin-8 (IL-8) and type I interferons (IFNs) in response to extracellular inflammatory stimulation signals (Kawai and Akira, 2007). Nod-like receptors (NLRs) also play an important role in the perception of inflammation or viral invasion. Oligonucleotide binding of NLRs to domain-containing protein 1 (NOD1) and NOD2 triggers a signaling cascade after ligand recognition that is similar to the cascade initiated by TLRs and which leads to the production of inflammatory cytokines (Kufer and Sansonetti, 2007). The other part of the NLR mediates the activation of caspase 1 which triggers caspase 1-dependent pyroptosis and releases inflammatory cytokines IL-18 and IL-1β (Martinon and Tschopp, 2007; Wang and Zhang, 2020). TLR, NOD1 and NOD2 coactivate caspase1 and produce large amounts of IL-1β (Kufer and Sansonetti, 2007).

### Research Related to Osteoarthritis and Pyroptosis

Currently, only a few studies have investigated the mechanism of interaction between OA and pyroptosis and most studies have focused on the role of the NLRP3 inflammasome (Table 1). NLRP3 inflammasomes have an important role in the pathogenesis of autoinflammation, cancer and degenerative diseases. In OA, perichondral synovial cells stimulated by DAMPs lead to the increased release of the NLRP3 inflammasomes, IL-1β and IL-18 on the cartilage surface to further exacerbate inflammatory cytokine production and promote pyroptosis (Man and Kanneganti, 2015). Shuya Wang et al. reported that activation of the AMPK signaling pathway by exogenous stromal cell-derived factor-1 (SDF-1) inhibits the NLRP3 inflammasome which in turn inhibits the scorching process of osteoarthritic synovial cells (Wang et al., 2021). P2X7R is a purinoceptor that is a non-selective cation channel gated by adenosine triphosphate. P2X7R mediates Na and Ca influx and K efflux, and is involved in a variety of inflammatory responses and different mechanisms of cell death (Surprenant et al., 1996; Bartlett et al., 2014). P2X7R participates in NLRP3 and caspase-11 distinct pathway-mediated pyroptosis and produces cartilage degrading enzymes to activate inflammatory factors in synovial tissue (Haseeb and Haqqi, 2013; Viganò and Mortellaro, 2013; Li et al., 2021a). Activated P2X7 promotes extracellular matrix degradation and pyroptotic inflammation in OA chondrocytes through NF-κB/NLRP3 crosstalk to aggravate the symptoms of OA (Li et al., 2021b).

**TABLE 1:** Summary of the main areas of research and potential applications of pyroptosis in osteoarthritis (Wang et al., 2021; Li et al., 2021a; Li C. et al., 2021; Liu et al., 2020; Yu et al., 2021; Yan et al., 2020; Hu et al., 2020; Zu et al., 2019; Zhao L. R. et al., 2018; Liu et al., 2019; Zhang and Xing, 2019; Xiao et al., 2021; Zhang et al., 2019b; Qian et al., 2021).

Important targets	Disease	Experimental subjects	Intervention factors	Cytokines	Biological function	Activation pathway
NLRP1, NLRP3	KOA	Human FLSs	**LPS**, ATP	IL-1β, uricacid, IL-18	Promote pyroptosis	Inflammasome, Caspase-l
NLRP3	OA	Male Wistar mice chondrocytes	**Icariin (ICA)**, LPS	MMP-1, MMP-13, NRLP3, IL-1β, IL-18, Col II	Inhibits pyroptosis	Inflammasome, Caspase-l
NRF2, NLRP3	OA	C57BL/6 male mice chondrocytes	**Lico A**, LPS	NLRP3, ASC, GSDMD, caspase-1, IL-1β, IL-18, Col II, aggrecan	Inhibits pyroptosis	NRF2/HO-1/NF-κB, Inflammasome, Caspase-l, p65, IκB-α
NLRP3	OA	C57BL/6 male mice	**Loganin**	MMP-3, MMP-13, Col II, Col X, CD31, cryopyrin, caspase-1, endomucin	Inhibits pyroptosis	NF-κB, Inflammasome, Caspase-l, p65, IκB-α
NLRP1, NLRP3	OA	C57BL/6J mice chondrocytes	**Morroniside,** DMM	MMP13, NLRP3, Caspase-1, Caspase-3, Ki67	Inhibits pyroptosis	NF-κB, Inflammasome, Caspase-l, p65, IκB-α
Hedgehog	OA	C57BL/6 male mice, Human chondrocyte cell (C28/I2)	**GANT-61**, Indomethacin, LPS	TNF-α, IL-2, IL-6, IL-1β, IL-18, caspase-1	Inhibits pyroptosis	Caspase-l, Hedgehog, Inflammasome
NLRP3	OA	C57BL/6 male mice, Human FLSs	**SDF-1** aka **CXCL12**	NLRP3, Caspase-1, ASC, IL-1β, GSDMD	Inhibits pyroptosis	AMPK, PI3K–mTOR, Caspase-l, Inflammasome
NLRP3	KOA	SD male rats, fibroblasts, synovial macrophage	**LPS**, ATP	IL-1β, IL-18, HMGB1, Caspase1, NLRP3, ASC, TGF-β, PLOD2, COL1A1, TIMP1, GSDMD	Inhibits pyroptosis	Inflammasome, Caspase-l
NLRP1, NLRP3	KOA	SD male rats, FLSs	**HMGB1**, LPS, ATP	IL-1β, HMGB1, Caspase1, NLRP3, NLRP1, GSDMD	Promote pyroptosis	Inflammasome, Caspase-l
NLRP1, NLRP3	KOA	SD female rats, FLSs	**HIF-1α**, LPS, ATP	IL-1β, IL-18, TGF-β, ASC, PLOD2, COL1A1, TIMP1, GSDMD, caspase-11	Promote pyroptosis	Inflammasome, Caspase-l
NLRP3	OA	SD rats, Chondrocytes	**USP7**, NOX4, H_2_O_2_	Caspase1, MMP-1, MMP-13, GSDMD, NLRP3	Promote pyroptosis	Inflammasome, Ubiquitinylation, Caspase-l, NOX4, ROS, NLPR3
NLRP3	OA	SD male rats, Chondrocytes	**P2X7 Receptor**, MIA, BzATP	MMP13, NF-κB, Col II, NLRP3, Caspase-1, p65, P2X7, IL-1β	Promote pyroptosis	NF-κB, NLPR3, Caspase-l, Inflammasome
NLRP1, NLRP3	OA	C28/I2 chondrocytes	**LPS**, ATP, Disulfiram, Glycyrrhizic acid	Caspase-1, GSDMD, NLRP3, IL-1β, IL-18, HMGB1	Inhibits pyroptosis	Inflammasome, Caspase-l, NLPR3
NLRP3, miR-107	KOA	Chondrocytes	**LPS**, ATP, miR-107	IL-1β, HMGB1, IL-18, Caspase-1, Col II, MMP13, GSDMD, TLR4	Inhibits pyroptosis	Inflammasome, Caspase-l, NLPR3

Recent studies have shown that the combined effect of disulfiram and glycyrrhizic acid at standard concentrations protects chondrocytes, inhibits the inflammatory responses and reduces pyroptosis (Li C. et al., 2021). Ubiquitin (Ub)-specific proteases (USPs), also known as deubiquitinating enzymes, remove Ub from Ub conjugates and regulate a variety of cellular processes (Ovaa et al., 2004). USP7 is a member of the ubiquitin-specific proteases. USP7 inhibitors attenuate H2O2-induced chondrocyte damage and pyroptosis by inhibiting the NOX4/NLRP3 signaling pathway (Liu et al., 2020).

The therapeutic role of Chinese medicine in the management of OA is gaining increasing attention. For example, Morroniside significantly inhibits the NF-κB signaling pathway, decreases the expression of NLRP3 and Caspase-1, and reduces the nuclear translocation of p65, thereby inhibiting the onset of pyroptosis and delaying the progression of OA (Yu et al., 2021). Chondrocyte pyroptosis is inhibited by licochalcone A (Lico A) through inhibition of the NLRP3 inflammasome (Yan et al., 2020). Jiaming Hu et al. reported that loganin ameliorates cartilage degeneration and the development of OA in a mouse model through inhibition of NF-κB activity and pyroptosis in chondrocytes (Hu et al., 2020). In animal models, Icariin (ICA) attenuates chondrocyte damage and OA by inhibiting the NLRP3 signaling-mediated caspase-1 pathway to reduce pyroptosis (Zu et al., 2019). NLRP1 and NLRP3 inflammasomes mediate the onset of pyroptosis in knee OA (KOA) via the Caspase-l/IL-1β inflammatory pathway (Zhao Y. et al., 2018). Shi et al. showed that increased lipopolysaccharide (LPS) and ATP in joint-space may promote KOA by NLRP3 Inflammasome (Shi et al., 2018). Overall, the role of inflammasomes such as NLRP3 and its regulators in pyroptosis suggests that NLRP3 may be a promising biomarker for the diagnosis and monitoring of OA. Therapeutic targeting of NLRP3 may be a potential strategy for the treatment of OA.

### Ferroptosis

Ferroptosis is a newly discovered form of regulated cell death that differs from the traditional cell death programs of necrosis, apoptosis, and pyroptosis that are caused by iron-dependent and lethal lipid peroxidation. Ferroptosis was first proposed by Dixon in 2012 (Dixon et al., 2012) and describes a form of cell death induced by the small molecule Erastin which inhibits cystine import leading to glutathione depletion and inactivation of the phospholipid peroxidase glutathione peroxidase 4 (Gpx4) (Yang et al., 2014; Stockwell et al., 2017). GPX4 converts potentially toxic lipid hydroperoxides (L-OOH) to non-toxic lipid alcohols (L-OH) (Ursini et al., 1982; Stockwell et al., 2017). Inactivation of Gpx4 by depletion of GSH with Erastin or with a direct Gpx4 inhibitor, (1S,3R)-RSL3, ultimately leads to overwhelming lipid peroxidation and cell death.

Inactivation of GPX4-RSL3 directly induces ferroptosis. Erastin and RSL3 were first identified as ferroptosis-inducing compounds (Dolma et al., 2003; Yang and Stockwell, 2008). Erastin inhibits the transfer of cysteine causing loss. Cysteine is an essential component of glutathione and so indirectly induces ferroptosis (Latunde-Dada, 2017). Ferroptosis can be suppressed by iron chelators, lipophilic antioxidants, inhibitors of lipid peroxidation, and depletion of polyunsaturated fatty acids. This process also correlates with the accumulation of markers of lipid peroxidation (Stockwell et al., 2017). Recent studies suggest that mobilization and upregulation of the transferrin receptor (TfR) can be a potential marker of iron death (Stockwell et al., 2017; Kajarabille and Latunde-Dada, 2019). In contrast to other forms of programmed cell death, ferroptosis exhibits specific morphological and biological features (Table2).

**TABLE 2:** The main morphological, biochemical, and signaling pathways involved in the regulation of pyroptosis, apoptosis, necroptosis, and ferroptosis (Kerr et al., 1972; Xu and Shi, 2007; Charlier et al., 2016; Newton and Manning, 2016; Xie et al., 2016; Yang and Stockwell, 2016; Zargarian et al., 2017; Jiang and Stockwell, 2021).

Cell Components/Events	Cell Death Types			
	**Pyroptosis**	**Apoptosis**	**Necroptosis**	**Ferroptosis**
Cell morphology	Gradual flattening	Shrinkage	Swelling	Smaller and rounder
Nucleus	Enrichment	Condensation and rupture	Nuclear condensation (pyknosis)	Normal nuclear size
Cytoplasm	Osmotic swelling	Retraction of pseudopods, reduction of cellular volume	Cytoplasmic swelling, swelling of cytoplasmic organelles	Mitochondrial membrane rupture and atrophy
Cell membrane	Formation of membrane pores, loss of integrity	Plasma membrane blebbing	Rupture of plasma membrane	Lack of rupture and blebbing of the plasma membrane
Chromatine	Random breakage degradation	Condensation	Fragmented	Lack of condensation
DNA	Random breakage degradation	Intranucleosomal cleavage-DNA laddering	Random cleavage DNA Smear	None
Lysosomial enzyme	Damage	Inside apoptotic bodies	Leakage	None
Special microstructure	Pyroptotic bodies	Apoptotic bodies	Necroptotic bodies	Mitochondrial membrane rupture
Inflammation	Yes	No	Yes	Yes
Key role	Caspase-1, Caspase-4/5/11	Caspase-3, Caspase-6, Caspase-7	RIP1, RIP3, TNF-α, Fas, Necrostatin-1, MLKL	GPX4, Phospholipid peroxidation, Iron
signal pathway	Gasdermin, NLRs	Fas-FasL, TRAIL-DR, TNFa-TNFR1, mitochondrial pathway	IKKα/IKKβ, NF-Κb, TNF-α	Mevalonate, AMPK, Hypoxia, glutathione depletion, Glutaminolysis, Transsulfuration, Heat shock protein beta 1
ATP requirement	Yes	Yes	No	Yes

### The Relationship Between Ferroptosis and Disease

The mechanisms of interaction between ferroptosis-inducing compounds and ferroptosis signaling pathways remain to be fully elucidated. A growing body of experimental evidence suggests that excessive iron contributes to oxidative tissue damage and organ dysfunction resulting in the development of cirrhosis (Yu et al., 2020), cardiomyopathy (Fang et al., 2020), diabetes and other diseases (Stockwell et al., 2020; Sha et al., 2021). In an animal model of traumatic brain injury (TBI), ferroptosis was shown to be involved in acute central nervous system (CNS) trauma based on glutathione peroxidase activity, lipid-responsive oxygen species and observations of mitochondrial shrinking (Xie et al., 2019). The characteristic products of ferroptosis have also been demonstrated in spinal cord injury and the source of iron is closely related to red blood cell rupture, hemolysis (Yao et al., 2019).

The role of ferroptosis in the treatment of hepatocellular carcinoma has also been a research focus. Urano et al. showed that the combination of iron inhibitors and anti-angiogenic drugs enhanced the tumor-killing effects of sorafenib by inducing cell cycle arrest and apoptosis (Urano et al., 2016). Studies from as early as 1992 have observed selective accumulation of iron in Aβ aggregation areas and neurofibrillary tangles in the brain in Alzheimer’s disease (Good et al., 1992). Ayton et al. showed that excessive accumulation of iron in the brains of Alzheimer’s patients may be associated with accelerated cognitive decompensation (Ayton et al., 2020).

**FIGURE 2:**
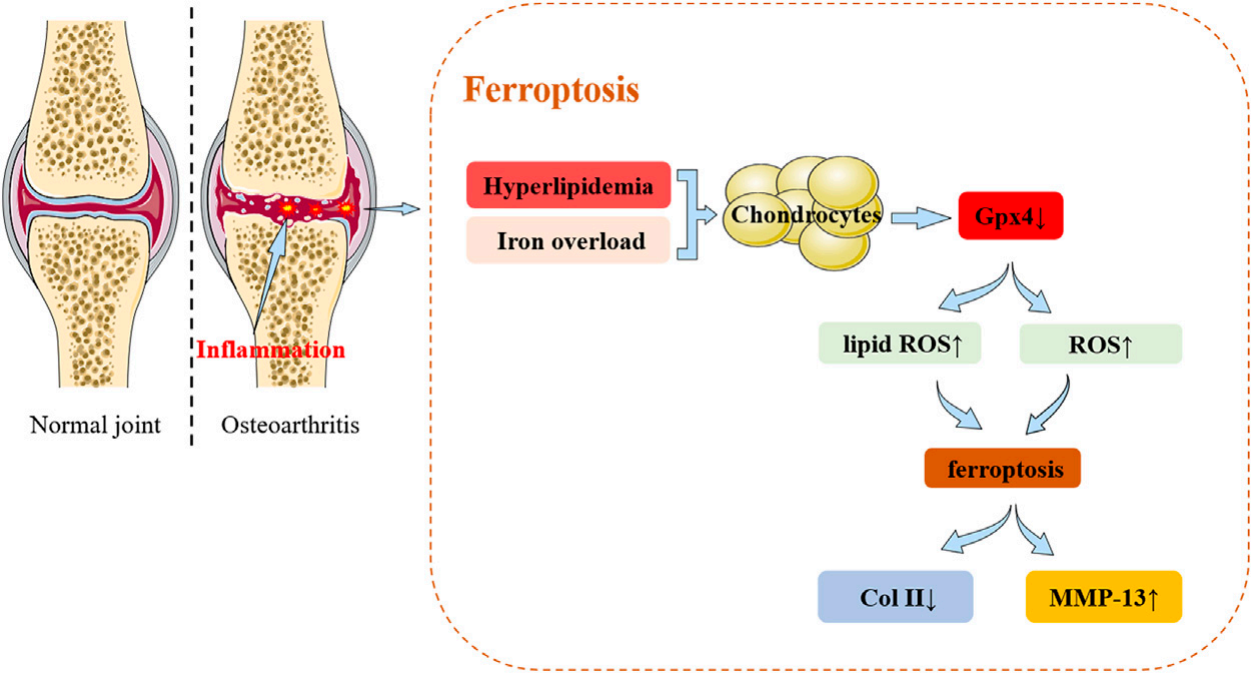
The relationship between ferroptosis and osteoarthritis (OA). Ferroptosis: In cellular environments stimulated by iron overload, hyperlipidemia, inflammation, the expression of Gpx4 in chondrocytes decreases. These changes lead to the accumulation of reactive oxygen species and lipid peroxides to ultimately induce ferroptosis. Ferroptosis in turn can progressively exacerbate the inflammatory response leading to the increased expression of MMP-13 and decreased expression of collagen II in chondrocytes to accelerate the progression of OA.

Currently, very few studies have focused on the role of ferroptosis in OA. Recent research findings from the past 2 years are summarized in Figure 2. Yao X et al. used chondrocytes extracted from the knee joints of C57BL/6J mice as an *in vitro* experimental model of OA using interleukin-1β and ferric ammonium citrate to mimic the inflammatory response and iron overload (Yao et al., 2021). The study demonstrated that ferrostatin-1 attenuates IL-1β and Fac induced cytotoxicity, the accumulation of reactive oxygen species (ROS) and lipid-ROS, and the expression of ferroptosis-related proteins to promote activation of the NRF2 antioxidant system. This was the first study to demonstrate that chondrocytes underwent ferroptosis *in vitro*.

In a lipopolysaccharide (LPS)-induced OA cell model, icariin (ICA) reduced the expression of iron transport factor TFR1. ICA activated the Xc-/Gpx4 axis to exert an inhibitory effect on the expression of Gpx4, SLC7A11 and SLC3A2L which acted to significantly reduce cell death in induced cells and inhibit ferroptosis (Luo and Zhang, 2021). Zhang et al. identified a new subpopulation of chondrocytes located in the nucleus pulposus of the intervertebral disc using single-cell RNA-seq analysis. The study revealed the presence of ferroptosis during disc degeneration (Zhang et al., 2021).

Ferroptosis has also been shown to have an important role in the development of cancer and inflammatory and chronic diseases. In OA, high concentrations of iron can promote joint degeneration and facilitate the development of OA. However, evidence from relevant studies is lacking and the detailed molecular mechanisms by which iron compromises to cartilage remains to be understood.

### Apoptosis

Apoptosis is the active, physiological process of cell death that is activated under specific physiological or pathological conditions. Apoptosis is regulated by intrinsic genetic mechanisms of autologous damage to the organism (Rathmell and Thompson, 2002). Apoptosis is regulated by apoptosis-related genes and so the process is also known as programmed cell death (PCD) or type I cell death.

The concept of apoptosis was first introduced by Kerr, Wyllie and Currie in 1972 (Kerr et al., 1972). “Apoptosis” is a Greek word meaning “to leave or fall” implying that apoptosis occurs when a cell dies similar to the natural withering of leaves or flowers. This metaphor emphasizes that apoptosis is an important part of the normal life cycle of an organism, and that it is an active process under strict genetic control. Apoptosis has a wide range of biological functions during the development and differentiation of the organisms, in immune regulation, and the maintenance of tissue stability. The process is also involved in the removal of redundant or harmful cells and the prevention of cancer (Honarpour et al., 2001; Luke et al., 2003; Ke et al., 2018).

Apoptosis is morphologically characterized by reductions in cell size, loss of connections, and detachment from surrounding cells. The cytoplasmic density gradually increases and the mitochondrial membrane potential disappears. During apoptosis, the nucleoplasm becomes concentrated in the nucleus, the nucleolus becomes broken, and DNA is degraded into 180–200 bp fragments (Enari et al., 1998). The entire cell has an intact cytosolic structure with vesicle formation which eventually divides and wraps the apoptotic cell into several apoptotic vesicles. This process does not involve the release of cellular contents and the inflammatory response is not activated (Mariño and Kroemer, 2013).

**FIGURE 3:**
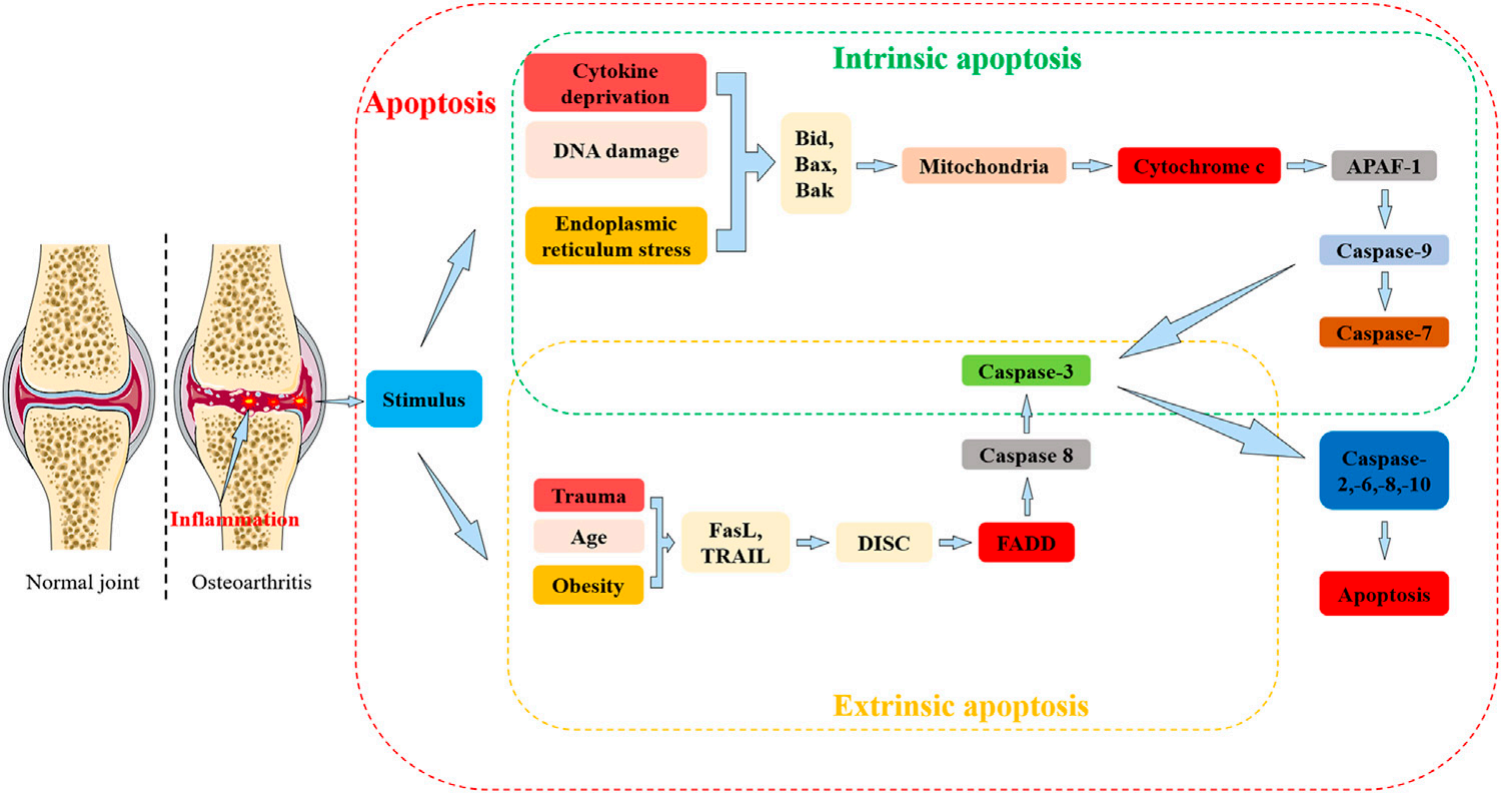
The relationship between apoptosis and osteoarthritis (OA). Apoptosis: Apoptosis is can be divided into extrinsic and intrinsic pathways. In the extrinsic pathway, death receptors (including Fas, TRAIL) are activated and bind to corresponding ligands (including FasL) in response to stimulation by risk factors for OA (including trauma, age, obesity, etc.) to form a multi-protein complex, also known as the DISC. Activation of Caspase-8 is mediated by FADD, and Caspase-8 further activates Caspase-3 to propagate apoptotic signals. In the intrinsic pathway, intracellular damage, DNA damage and endoplasmic reticulum stress lead to activation mainly in the mitochondria and endoplasmic reticulum BCL2 protein family members (including Bid, Bax, and Bak). Bcl2 protein family members can activate cytochrome c which can further activate apoptosis protease activating factor-1 (APAF-1) and caspase-9. Activated caspase-9 further activates caspases-3 and -7 which in turn activate caspases-2,-6,-8,-10 to create a positive feedback loop that amplifies the apoptotic signal and induces apoptosis.

Apoptosis can be defined as either extrinsic or intrinsic depending on the stimulus (Figure 3). Extrinsic apoptosis is also known as the death receptor apoptotic pathway and intrinsic apoptosis refers to the mitochondrial apoptotic pathway (Elmore, 2007). Death receptors refer to Fas (also known as DR2, APO-1, or CD95), tumor necrosis factor receptor 1 (TNFR1) or tumor necrosis factor-associated apoptosis-inducing ligand receptor (TRAILR), all of which are members of the Tumor Necrosis Factor Receptor Superfamily (TNFRSF) (Lavrik et al., 2005).

Death receptors are transmembrane glycoproteins located on the surface of cell membranes that cause conformational changes in cell membrane delivery when receiving death ligands from extrinsic apoptotic signaling pathways and rapidly activate caspase ultimately leading to apoptosis (Mariño and Kroemer, 2013). The engagement of the death receptor, Fas, binds to its ligand FasL to assembly a typical multiprotein complex called the death-inducing signaling complex (DISC). DISC formation allows the recruitment and activation of initiator caspase-8, mediated by the Fas-associated protein with death domain (FADD) adaptor molecule (Charlier et al., 2016). The recruitment of caspase-8 leads to autoproteolytic cleavage and subsequent activation (Medema et al., 1997; Charlier et al., 2016). The active fragment of caspase-8 propagates apoptotic signals by activating caspase-3 fragment activity which further catabolizes cellular components (Fuentes-Prior and Salvesen, 2004).

Fas is involved in regulating the DISC-mediated production of active caspase-8 and activates caspase-9 prior to activation of caspase-3 (Scaffidi et al., 1998). Intrinsic apoptosis triggers include intracellular damage, cytokine withdrawal, DNA damage, oxidative or endoplasmic reticulum (ER) stress, and cytosolic Ca^2+^ overload (Vanden Berghe et al., 2015). These stimuli ultimately activate members of the BCL2 family of proteins located primarily in the mitochondria and endoplasmic reticulum and have contrasting effects on cell fate. For example, Bcl-xs (Boise et al., 1993), Bcl-GL (Guo et al., 2001), Bok (Hsu and Hsueh, 1998), Bax (Oltvai et al., 1993), and Bak can promote apoptosis (Chittenden et al., 1995), whilst A1 (Lin et al., 1996), Mcl-1 (Kozopas et al., 1993), Bcl-B (Ke et al., 2001), Bcl-2 (Tsujimoto et al., 1985), Bcl-x (Boise et al., 1993), and Bcl-w can prevent apoptosis (Gibson et al., 1996; Kroemer and Reed, 2000).

The proteins that lead to apoptosis can be functionally classified based on whether they can function independently of caspases (i.e., Omi/HtrA2, apoptosis-inducing factor AIF) or activate caspases either directly or indirectly (e.g., Smac/DIABLO, cytochrome c) (van Loo et al., 2002a; van Loo et al., 2002b; Charlier et al., 2016). Apoptosis protease-activating factor-1 (APAF-1) is a key component of the apoptosome (Green, 2003). Cytochrome c can activate APAF-1 whilst forming an activation platform for the caspase-9 promoter in the mitochondrial pathway by oligomerization with APAF-1 (Bao and Shi, 2007). Activated caspase-9 further activates caspases-3 and -7, which in turn activate caspases-2,-6,-8,-10 forming a positive feedback loop that amplifies apoptosis signals and induces apoptosis (Slee et al., 1999; Van de Craen et al., 1999).

### Signaling Pathways Involved in Osteoarthritis and Apoptosis

Recent studies focused on apoptosis of articular chondrocytes in OA involve the inflammatory response, signaling pathways and target modulation. Interleukin-1β (IL-1β) is an important inflammatory factor that belongs to the interleukin-1 (IL-1) family and plays a key role in the pathogenesis of OA (López-Armada et al., 2006; Chevalier et al., 2011). IL-1β increases apoptosis in articular chondrocytes by stimulating the expression of TNF, Fas-associated death region protein, and Caspases-3, and -8 (Qin et al., 2013).

Current research has focused on the role of non-coding RNAs regulating IL-1β-mediated apoptosis in chondrocytes. Recent studies demonstrate that the LINC00623/miR-101/HRAS axis modulates chondrocyte apoptosis, senescence and extracellular matrix (ECM) degradation in OA through MAPK signaling (Lü et al., 2020). Also, it has been shown that miR-27a is a regulator of the PI3K-Akt-mTOR axis in human chondrocytes that could be involved in OA (Cai et al., 2019). Junkui Xu et al. reported that LncRNA SNHG7 alleviates IL-1β-induced OA by inhibiting miR-214-5p-mediated PPARGC1B signaling pathways (Xu et al., 2021).

The tumor necrosis factor (TNF) superfamily is a group of cytokines produced by a variety of cell types including macrophages (Baud and Karin, 2001; Wajant et al., 2003). TNF plays a key role in immunity, inflammation, and the control of cell differentiation, proliferation, and apoptosis (Wajant et al., 2003). TNF-α is one of the most important signaling molecules in this family. TNF-R1 mediates most of the biological functions of TNF-α and contains a death structural domain. TNF-R1 can induce apoptosis by activating the NF-κB, JNK and MAPKs signaling pathways (Chen and Goeddel, 2002).

The combination of long-stranded non-coding RNA (LncRNA) and microRNA (MiRNA) has gradually replaced single, localized research approaches as the main approach to study the roles of these molecules in OA today. Xu Kai et al. reported that LncRNA PVT1 induces chondrocyte apoptosis through upregulation of TNF-α in synoviocytes by sponging miR-211-3p (Xu K. et al., 2020). Wang Yingjie et al. reported that MiR-140-5p inhibits the PI3K/AKT signaling pathway and suppresses the progression of OA by targeting HMGB1 (Wang et al., 2020).

Mitogen-activated protein kinases (MAPKs) are a class of serine/threonine protein kinases that are widely present in eukaryotic cells. MAPKs are activated by extracellular signals, physical stimuli, and inflammatory cytokines. They also regulate the activity of transcription factors to control the expression of related genes and elicit cellular responses (Wang et al., 2016). The MAPK subfamily includes p38mapk, extracellular regulated protein kinases (ERK) and c-Jun N-terminal kinase (JNK) (Davis, 2000; Tang et al., 2012). JNK can promote the expression of apoptotic genes by phosphorylating c-jun and by increasing the expression of proteins related to the Fas/FasL signaling pathway. Conversely, JNK induces phosphorylation and inactivation of related anti-apoptotic proteins to promote apoptosis in OA (Shajahan et al., 2012; Zhou et al., 2019). Fas signaling can initiate apoptosis by activating caspase-8 which in turn can activate the downstream effector caspase-3 (Xue et al., 2017).

Notch signaling is an evolutionarily conserved pathway that plays an important regulatory role in cell fate determination, proliferation, differentiation, and dynamic homeostasis. The Notch signaling pathway also plays an important role in proliferation and differentiation during chondrogenesis and in the development of cartilage (Kohn et al., 2012; Mirando et al., 2013). The Notch pathway consists primarily of Notch receptors, Notch ligands, and downstream target genes. In post-traumatic OA, the Notch pathway is highly activated in human and mouse joint tissues (Karlsson et al., 2008). Notch1, JAG1 and other downstream target genes are overexpressed in OA tissue biopsies (Karlsson et al., 2008). However, in a mouse model, loss of Notch signaling in OA indicated an important role in maintaining osteoarticular cartilage growth (Liu et al., 2015). Also, intra-articular injection of the Notch complex inhibits cartilage degeneration in a mouse OA model (Hosaka et al., 2013). Overall, a dual role for Notch signaling in maintaining the normal physiological function of articular cartilage and promoting the progression of OA has been observed. In short, Notch signaling plays a complex role in cartilage homeostasis and transient or physiological Notch signaling in chondrocytes favors a balanced anabolic and catabolic response. In contrast, sustained or enhanced Notch activity elicits a pathological response through the simultaneous suppression of chondrogenic genes and the induction of genes encoding catabolic factors (Zieba et al., 2020).

Melatonin (N-acetyl-5-methoxytryptamine) is a molecule that is produced primarily by the pineal gland and other organs that acts to reduce peroxidative damage in the body (Acuña-Castroviejo et al., 2014; Reiter et al., 2016). Melatonin plays an important role in inflammation, apoptosis, proliferation, and metastasis. It has also been shown to have a protective role in chronic diseases including OA, osteoporosis, COVID-19, Parkinson’s, Alzheimer’s, cancer, and sepsis (Zhang R. et al., 2020; Luo et al., 2020; Xu et al., 2018; Hosseinzadeh et al., 2016).

Melatonin prevents apoptosis and promotes cell survival by inhibiting p38, phosphorylation of JNK MAPKs, and p53 activation by limiting cytochrome c release and activating procaspases proteases (Tomás-Zapico and Coto-Montes, 2005). LIM et al. investigated the effects of melatonin on human chondrocytes and rabbit OA models (Lim et al., 2012). The study showed that melatonin inhibits H_2_O_2_-induced cytotoxicity, suppresses the production of NO and PEG2 production, and blocks the H_2_O_2_-induced release of TNF- α, IL- 1β, and IL- 8. Animal experiments have shown that intra-articular injection of melatonin protects articular cartilage by targeting miR-140. This acts to prevent the disruption of cartilage matrix homeostasis and slows the progression of surgically induced OA in mice (Zhang Y. et al., 2019).

Currently, apoptosis is an important focus of OA research as an increasing number of signaling pathways have been shown to be involved. The interaction between non-coding RNA and OA is subject to ongoing investigations. Further elucidation of the respective roles of apoptosis and non-coding RNAs may facilitate the development of novel gene therapy and targeted approaches for the treatment of OA.

### Necroptosis

It was more than 2 centuries ago that pathologists determined that necrosis was a cause and consequence of disease (Linkermann, 2014). 100 years later, apoptosis was first discovered. Previous studies identified the pathophysiological importance of necroptosis in myocardial infarction and stroke (Smith et al., 2007), atherosclerosis (Lin et al., 2013), ischemia-reperfusion injury (Linkermann et al., 2012), pancreatitis (Wu et al., 2013), inflammatory bowel disease (Welz et al., 2011), and several other common clinical disorders (Linkermann, 2014).

Necrosis has long been described as the exposure of cells to extreme physicochemical stresses resulting in rapid cell death. However, necrosis can be induced under different stimulatory conditions (e.g., inflammatory factors, interferon-g (IFN-g), ATP depletion, ischemia-reperfusion injury, and pathogens) with steps and signaling events similar to the cell death program (Vanlangenakker et al., 2012; Kaczmarek et al., 2013). This process of regulated necrosis is referred to as necroptosis. Morphological changes during necroptosis include a translucent cytoplasm, swelling of the organelles, permeabilization of the lysosomal and plasma membranes, increased cell volume (oncosis), and mild chromatin condensation (Pasparakis and Vandenabeele, 2015) (Table 2).

In contrast to apoptosis and pyroptosis, necroptosis is a caspase-independent death program. Necroptosis is programmed necrotic cell death caused by RIP1/RIP3 and MLKL under various pathological conditions (Han et al., 2011; Chan et al., 2015; Yoon et al., 2017). Apoptosis and necroptosis are closely related, and TNF can determine the final fate of cells (Van Herreweghe et al., 2010). Once TNF binds to TNF receptor 1, TNF induces receptor trimerization and recruits the death domain (DD)-containing adaptor proteins TRADD, TRAF2, and RIP1 to form the so-called complex I (Zhang et al., 2018). Several components of complex I recombine to form a cytosolic complex (complex II) that recruits FADD (Fas-associated via DD) via DD-mediated interactions. In complex II, FADD recruits procaspase-8, whilst RIP1 recruits RIP3. When RIP3 is absent or present at low levels, caspase-8 can activate automatically and the cell undergoes apoptosis (Zhang et al., 2018).

In contrast, in the presence of high concentrations of RIP3, complex II tends to recruit large amounts of this protein and turns itself into a so-called necrosome (Silke et al., 2015). Procaspase-8 in the necrosome cleaves RIP1 and RIP3 preventing the initiation of the necroptosis (Zhang et al., 2018). Therefore, the development of necroptosis requires inhibition of caspase-8 activity (Zhang et al., 2009). RIP3 can be auto-phosphorylated in response to homo-interactions (Chen et al., 2013). Auto-phosphorylated RIP3 recruits and phosphorylates the mixed-lineage kinase domain-like protein (MLKL) (Sun et al., 2012). Phosphorylated RIP3 recruits and phosphorylates MLKL leading to MLKL oligomerization and translocation to the plasma membrane. MLKL oligomers execute necroptosis by generating cation channels causing plasma membrane rupture (Zhang et al., 2018). Recent studies have identified MLKL as the most important working protein for plasma membrane rupture (Chen et al., 2014).

In summary, RIP1 deubiquitination is critical for necrosome assembly and activation (Hitomi et al., 2008), whilst RIP3 determines the susceptibility of cells to move towards necroptosis (Linkermann, 2014) which is ultimately executed by phosphorylated MLKL (Cai et al., 2014).

### Necroptosis and Orthopaedic-Related Diseases

Emerging studies have focused on the link between necrosis and orthopaedic-related diseases. Osteoporosis is a systemic bone disease characterized by low bone mass and degenerative damage to the ultrastructure of bone trabeculae resulting in increased bone fragility and susceptibility to fractures (Ensrud and Crandall, 2017). In 2016, Cui et al. constructed an ovariectomy-induced osteoporosis rat model and found that the levels of RIP1, RIP3, and MLKL proteins were significantly elevated in rat femurs and a large number of necrotic osteocytes with positive TUNEL staining but negative caspase-3 staining were seen. The study also showed that administration of necrostatin-1 (Nec-1) significantly reduced the expression of RIP1, RIP3 levels and inhibited programmed necrosis of osteoblasts to reverse bone loss (Cui et al., 2016a; Cui et al., 2016b). Studies have found that excessive alcohol consumption leads to activation of RIPK1/RIPK3/MLKL signaling which increases necrotrophic apoptosis of osteoblasts and reduces osteogenic differentiation and bone formation *in vivo* and *in vitro*, leading to the development of osteoporosis (Guo et al., 2021).

Lumbar disc herniation (LDH) is a syndrome in which the lumbar disc degenerates and the nucleus pulposus protrudes outwards either alone or together with the fibrous ring and cartilage endplates. This causes irritation or compression of the sinus nerve and nerve roots with lumbar and leg pain as the main symptom (Deyo and Mirza, 2016). The pathogenesis of lumbar disc herniation includes disc degeneration, mechanical stress injury, immune inflammation, and imbalance of extracellular matrix metabolism (Deyo and Mirza, 2016). Necroptosis and lumbar disc herniation are closely associated processes. Recent studies have shown that necrosulfonamide (NSA) protects intervertebral disc degeneration via necroptosis and apoptosis inhibition (Zhang QX. et al., 2020). Chen et al. demonstrated in an *in vivo* model that RIPK1-mediated mitochondrial dysfunction and oxidative stress play a crucial role in NP cell necroptosis and apoptosis during compression injury. The synergistic regulation of necroptosis and apoptosis may exert more beneficial effects on NP cell survival to ultimately delay or prevent intervertebral disc degeneration (Chen et al., 2018).

**FIGURE 4:**
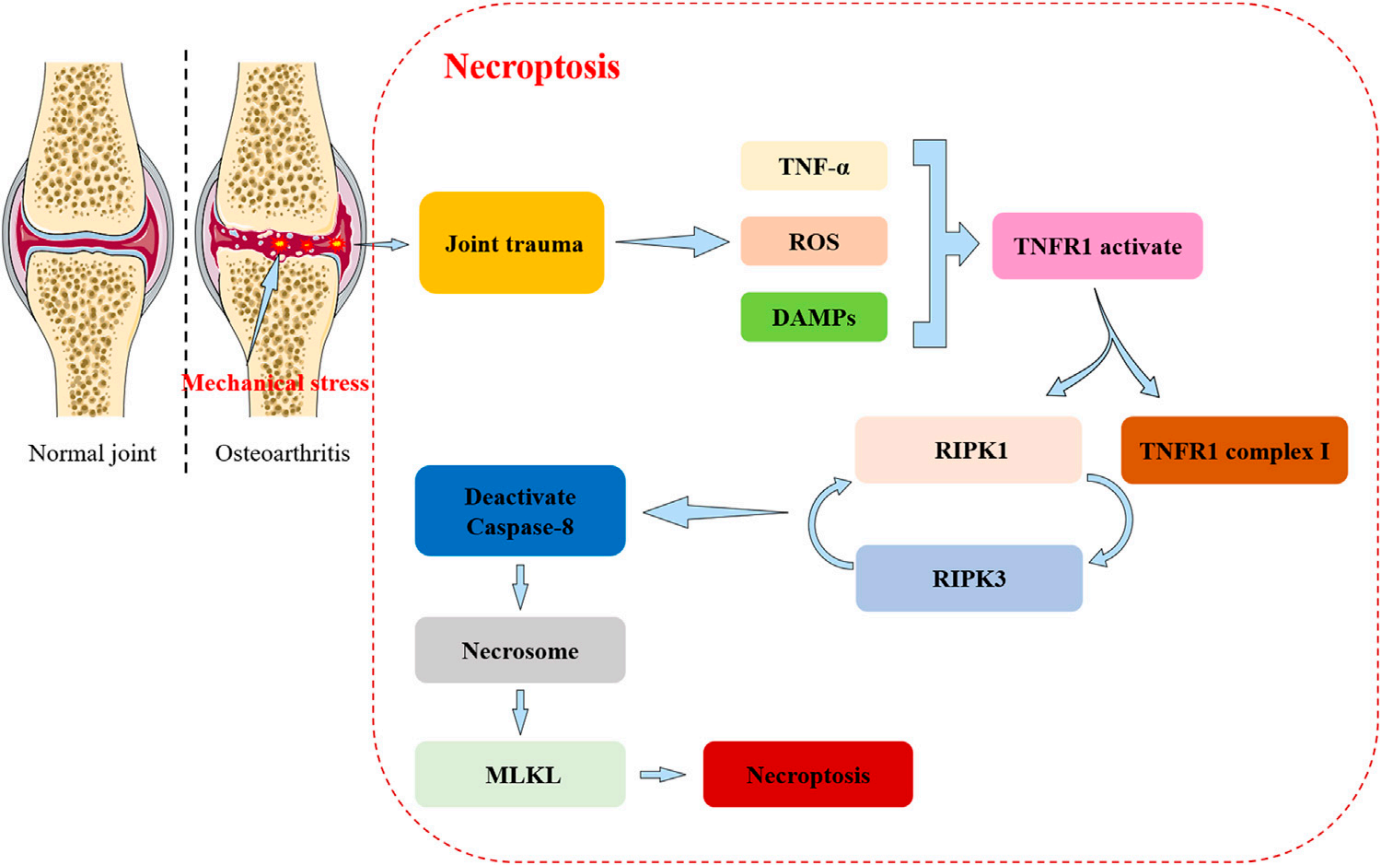
The relationship between necroptosis and osteoarthritis (OA). Necroptosis: Joint trauma leads to the release of death-related triggers including tumor necrosis factor alpha (TNF-α), reactive oxygen/nitrogen species (ROS/RNS), and damage associated molecular patterns (DAMPs). These factors subsequently lead to TNF receptor 1 (TNFR1) activation, forming the TNFR1 complex I and the receptor interacting protein kinase 1 (RIPK1). RIPK1 and RIPK3 interact to activate caspase-8. When caspase8 is inactivated it leads to the appearance of necrosomes which cause the recruitment and phosphorylation of mixed-lineage kinase domain-like protein (MLKL), ultimately leading to disruption of membrane integrity. This leads to the formation of necroptosis.

Research on the role and mechanisms of necroptosis in OA is becoming an area of increasing interest. Recent evidence suggests that oxidative and mechanical stresses can contribute to the development of necroptosis in OA (Riegger and Brenner, 2019) (Figure 4). Mechanical stress also mediates apoptosis and necroptosis in mandibular cartilage via RIP1 (Zhang et al., 2017). Cheng et al. demonstrate that upregulation of RIP1 contributions to OA pathogenesis by mediating chondrocyte necroptosis and ECM destruction via BMP7, a newly identified downstream target of RIP1, in addition to MLKL (Cheng et al., 2021). Chen et al. reported that PLCγ1 inhibition combined with inhibition of apoptosis and necroptosis increases cartilage matrix synthesis in IL-1β treated rat chondrocytes (Chen et al., 2021). Also, recent evidence has shown that perturbation of the TRIM24-RIP3 axis regulates mouse osteoarticular pathogenesis by activating RIP3 kinase and regulating the expression of catabolic factors (Jeon et al., 2020).

In summary, the development and progression of OA is closely related to RIP1 and RIP3. Recent studies have suggested that necroptosis has an important position in inflammation-related diseases. Necroptosis and apoptosis are closely related highlighting the complexity and diversity of mechanisms of disease.

## Discussion and Perspectives

OA is a complex chronic disease that is affected by age, gender, weight, mechanical injury and joint deformity. The number of people affected and the cost of medical treatment are increasing every year, hence there is a need for improved OA treatment options. The current pharmacological approach to treating OA is mostly palliative and surgery remains the ultimate option for patients. As the understanding of the etiology and pathogenesis of OA improves, more and more potential targets are being used to prevent the development and progression of the disease. The relationship between the pathogenesis of OA and different cell death types is likely to remain a future research focus.

During the development of OA, inflammatory mediators such as ROS, interleukins, NO, and MMP are closely related to chondrocyte apoptosis that involves the mitochondrial, death receptor and JNK signaling pathways. These signaling pathways are directly related to apoptosis in chondrocytes and regulate gene targets, proteins and miRNAs (Table 3).

**TABLE 3:** Summary of the pathogenesis of osteoarthritis (OA) and the relationship with different types of cell death (Hunter and Bierma-Zeinstra, 2019; An et al., 2020; Riegger and Brenner, 2019; Shi et al., 2019; Royce et al., 2019; Zhou et al., 2021; Yao et al., 2021; Li et al., 2020; Tang et al., 2018; Xu et al., 2019; Sun et al., 2021; Xu L. et al., 2020; Tian et al., 2020; Aluganti Narasimhulu and Singla, 2021; Zu et al., 2019; Zhao Y. et al., 2018).

Osteoarthritis pathogenesis	Cell Death Types			
	**Pyroptosis**	**Apoptosis**	**Necroptosis**	**Ferroptosis**
Increased inflammatory component	IL-1β↑, IL-18↑, Caspase-1↑, NLRP3↑, MMP-1↑, MMP-13↑, NLRP3↑, NLRP1↑	IL-1β↑, IL-6↑, IL-8↑, TNF-α↑, Bax↑, Bcl-2↑, ROS↑, MMP2↑, MMP9↑	MLKL↑, Cleaved caspase8↑, *p*-MLKL↑	Expression of catabolic genes Mmp3↑, Mmp13↑, Adamts5↑, Ptsg2↑, Col10a1↑
Mechanical overload	IL-1β↑, IL-18↑, Caspase-1↑, NLRP3↑, MMP-1↑, MMP-13↑	Cleaved caspase-3, -6, -7, and -8↑, actin polymerization↑	RIP1↑, RIP3↑, Caspase-8↑, ROS↑, Mitochondrial membrane potential↓	MMP13↑, collagen II↓
Metabolic alterations	Caspase-1↑, IL-1β↑, IL-18↑, Gasdermin-D↑	Phospho-fructose kinase 1 (Pfk1) l↓, hexokinase II (Hk2) l↓, ATP l↓, mitochondrial fusion	Inhibits necroptosis through the hypermethylation of the promoter	Reactive oxygen species (ROS) ↑, lipid ROS↑, MMP13↑, collagen II↓
Cell senescence	IL-1β↑, IL-18↑, activation caspase-1 or caspase-11	COL10A1↑, IL-1↑, TNF-α↑, MMP-13↑, ADAMTS5↑, COL2A1↓	Oxidative stress↑, mTOR signaling↑, DAMPs↑	Mmp3↑, Mmp13↑, Adamts5↑, Ptsg2↑, Col10a1↑

The relationship between pyroptosis and OA has recently attracted attention. Pyroptosis produced during cell scorch death promotes the development of OA which contributes to pain associated with OA. The current focus of research remains on NLRP3 inflammatory vesicles. Undeniably, current experimental evidence *in vivo* and *in vitro* strongly suggests a close relationship between pyroptosis and OA. However, the molecular regulatory mechanisms of the relevant signaling pathways and the interconnections between these factors have not been fully elucidated. Few studies have reported on the link between pyroptosis, non-coding RNAs and OA.

Ferroptosis is an iron-dependent, non-apoptotic form of cell death that is distinct from apoptosis, pyroptosis, or necrosis. The main features of ferroptosis are lipid peroxidation and iron overload. New mechanisms and novel targets have been identified in studies of tumors (including hepatocellular carcinoma, pancreatic cancer, breast cancer, renal clear cell carcinoma), neurological diseases (including Alzheimer’s disease, Parkinson’s disease), cardiovascular diseases (including myocardial injury, ischemia-reperfusion, hemorrhagic stroke), and chronic diseases (including OA, rheumatoid arthritis). This provides novel perspectives and strategies for the treatment of related diseases. The occurrence of ferroptosis involves the expression and regulation of multiple genes, with complex signaling pathways and mechanisms that have not been fully elucidated. Currently available studies cannot fully reveal the relationship between ferroptosis and disease, and more research is needed in this area.

In conclusion, apoptosis, ferroptosis and pyroptosis have important roles in the development of OA, but deeper studies are needed. Exploring the relationship between OA and cell death can provide a theoretical basis and enable the development of translational strategies towards curing OA Kayagaki et al., 2011.

## Glossary

OA: Osteoarthritis
KOA: Knee osteoarthritis
DAMPs: Damage associated molecular pattern molecules
TLRs: Toll-like receptors
PRRs: Particular pattern recognition receptors
ASC: Apoptosis-associated speck-like protein containing a CARD
FADD: Fas-associated protein with death domain
DISC: Death-inducing signaling complex
NF-κB: Nuclear factor-κB
MAPK: Mitogen-activated protein kinase
FLS: Fibroblast-like synoviocytes
NLRP: Nod-like receptor protein
ASC: Apoptosis-associated speck-like protein with a caspase-recruitment domain
GSDMD: Gasdermin D
LPS: Lipopolysaccharide
Lico A: Licochalcone A
SDF-1: Stromal cell-derived factor-1
ATP: Adenosine triphosphate
MMP-1: Matrix metalloproteinases 1
MMP-13: Matrix metalloproteinases 13
MMP-3: Matrix metalloproteinases 3
TGF-β: Transforming growth factor-β
PLOD2: Procollagen-lysine, 2-oxoglutarate 5-dioxygenase 2
COL1A1: Collagen type Iα1 chain
TIMP1: Tissue inhibitor of metalloproteinase 1.
DMM: Destabilization of the medial meniscus
HMGB1: High mobility group box 1
SD rats: Sprague-dawley rats
Human FLSs: Human fibroblast-like synoviocytes
HIF-1α: Hypoxia-inducible factor-1α
USP7: Ubiquitin-specific protease 7
ROS: Reactive oxygen species
Lipid-ROS: Lipid reactive oxygen species
NOXs: NAD(P)H oxidases
NOX4: One of the members of the NOX family
Gpx4: Glutathione peroxidase 4
H2O2: Hydrogen peroxide
MIA: Monosodium iodoacetate
BzATP: An ATP analog with greater potency than ATP
Col II: Collagen II
Col X: Collagen X
CNS trauma: Central nervous system trauma.
RIP1: Receptor interacting protein kinase-1
RIP3: Receptor interacting protein kinase-3
MLKL: Mixed-lineage kinase domain-like protein
TRADD: TNF-receptor associated via DD
TRAF2: TNF receptor-associated factor 2
